# Risk of Long-Term Ischemic Stroke in Patients With Traumatic Brain Injury and Incident Hypertension

**DOI:** 10.1089/neur.2024.0015

**Published:** 2024-04-22

**Authors:** Farid Radmanesh, Saef Izzy, Ran S. Rotem, Zabreen Tahir, Quinn J. Rademaker, Taha Yahya, Ahmad Mashlah, Herman A. Taylor, Marc G. Weisskopf, Ross D. Zafonte, Aaron L. Baggish, Rachel Grashow

**Affiliations:** ^1^ Divisions of Stroke, Cerebrovascular and Critical Care Neurology, Department of Neurology, Brigham and Women's Hospital, Boston, Massachusetts, USA.; ^2^ Division of Neurocritical Care, Department of Neurology, University of New Mexico, Albuquerque, New Mexico, USA.; ^3^ Football Players Health Study at Harvard University, Boston, Massachusetts, USA.; ^4^ Department of Environmental Health, Harvard T.H. Chan School of Public Health, Boston, Massachusetts, USA.; ^5^ Department of Neurology, Houston Methodist Hospital, Houston, Texas, USA.; ^6^ Morehouse School of Medicine, Atlanta, Georgia, USA.; ^7^ Department of Physical Medicine and Rehabilitation, Massachusetts General Hospital, Brigham and Women's Hospital, Harvard Medical School, Boston, Massachusetts, USA.; ^8^ Spaulding Rehabilitation Hospital, Charlestown, Massachusetts, USA.; ^9^ Institute for Sport Science and Department of Cardiology, Lausanne University Hospital, Lausanne, Switzerland.

**Keywords:** hypertension, stroke, traumatic brain injury

## Abstract

Traumatic brain injury (TBI) is independently associated with hypertension and ischemic stroke. The goal of this study was to determine the interplay between TBI and incident hypertension in the occurrence of post-TBI stroke. This prospective study used a hospital-based registry to identify patients without pre-existing comorbidities. TBI patients (*n* = 3664) were frequency matched on age, sex, and race to non-TBI patients (*n* = 1848). Follow-up started 6 months post-TBI or study entry and extended up to 10 years. To examine hypertension's role in post-TBI stroke, we used logistic regression models to calculate the effect estimates for stroke in four exposure categories that included TBI or hypertension in isolation and in combination. Second, we calculated the conditional direct effect (CDE) of TBI in models that considered hypertension as intermediary. Third, we examined whether TBI effect was modified by antihypertensive medication use. The 10-year cumulative incidence of stroke was higher in the TBI group (4.7%) than the non-TBI group (1.3%; *p* < 0.001). TBI patients who developed hypertension had the highest risk of stroke (odds ratio [OR] = 4.83, 95% confidence interval [CI] = 2.53–9.23, *p* < 0.001). The combined effect estimates were less than additive, suggesting an overlapping biological pathway. The total effect of TBI (OR = 3.16, 95% CI = 1.94–5.16, *p* < 0.001) was higher than the CDE that accounted for hypertension (OR = 2.45, 95% CI = 0.93–6.47, *p* = 0.06). Antihypertensives attenuated the TBI effect, suggesting that the TBI effect on stroke is partially mediated through hypertension. TBI is an independent risk factor for long-term stroke, and the underlying biological pathway may partly operate through TBI-precipitated hypertension. These findings suggest that screening for hypertension may mitigate stroke risk in TBI.

## Introduction

Traumatic brain injury (TBI) and hypertension are independent risk factors for ischemic stroke in the general population.^1,2^ Recent data from general populations, American-style football players, and military veterans suggest associations between TBI and hypertension, ^2–5^ although most studies included patients with pre-existing comorbidities.^1^ At present, the interplay between TBI, hypertension, and ischemic stroke, particularly in patients without pre-existing comorbidities that preceded TBI onset, is unknown. In this study, we examined the association between TBI and stroke in patients who developed or did not develop hypertension after TBI and who did not otherwise have other past neurological, cardiovascular, metabolic, endocrine, or psychiatric comorbidities. Among patients who developed hypertension, we also evaluated whether use of antihypertensive medications attenuated the association between TBI and stroke.

## Methods

The Mass General Brigham Research Patient Data Registry (RPDR) holds prospective inpatient, outpatient, and emergency department data.^2^ RPDR was used to identify TBI and non-TBI groups with equivalent distributions of age (range = 18–85 years), sex, and race. TBI patients (*N* = 3664) presented with TBI between 2000 and 2018 and had ≥4 years of follow-up after inclusion. Non-TBI patients (*N* = 1848) were those who had a visit for any non-trauma-related condition during the study period. Participants with known past comorbidities were excluded. All diagnoses were ascertained using the International Classification of Diseases, Ninth and Tenth Revisions (ICD-9/10; Supplementary Table S1).^3^ The Mass General Brigham Institutional Review Board approved this study.

The index date was defined as the date of TBI or when the non-TBI participants presented with a non-trauma condition. All patients diagnosed with hypertension or ischemic stroke within the first 6 months after the index date were removed to exclude prevalent hypertension or ischemic stroke in the acute phase of TBI. To allow for adequate time for ischemic stroke manifestation, we excluded patients with <4 years of follow-up, based on the median number of years from the index date to ischemic stroke in the entire study population. Diagnoses for ischemic stroke and hypertension were made at out- or inpatient follow-up encounters.

We used logistic regression to investigate the interplay between TBI, post-TBI hypertension, and ischemic stroke, adjusted for age, sex, race, and average yearly clinical visits during follow-up.^4^ First, to examine the individual and combined effect of TBI and incident hypertension on ischemic stroke, we defined four mutually exclusive exposure categories: 1) neither TBI nor hypertension (reference); 2) non-TBI with hypertension; 3) TBI without hypertension; and 4) TBI with hypertension. If TBI and incident hypertension have independent effects on ischemic stroke, the combined effect of both exposures should sum to the individual effects. However, if the pathways linking TBI and incident hypertension with ischemic stroke overlap, the combined effect would be less than the additive effect.

Second, we compared effect estimates for TBI in a model that did not consider hypertension (i.e., the full effect), with the conditional direct effect (CDE) estimate for TBI in a model that accounted for hypertension. Assuming no unmeasured confounding, a smaller CDE for TBI compared to the full effect could suggest that part of the effect of TBI on ischemic stroke operates through incident hypertension. Third, given the overall low prevalence of incident hypertension in the study cohort, we assessed whether the association between TBI and ischemic stroke in the subset of patients with hypertension differed by use of antihypertensive pharmacotherapy. If TBI and incident hypertension have overlapping pathways to ischemic stroke, one may expect that effective use of antihypertensive medications would attenuate the TBI effect. Analyses were performed using R software (4.2.2),^5^ with alpha defined as *p* < 0.05.

## Results

TBI and non-TBI groups shared similar demographic distributions (Table 1). Median follow-up time, mean age at diagnosis of ischemic stroke, and mean time from the index date to ischemic stroke were also comparable. The 10-year cumulative incidence of ischemic stroke in TBI (4.7%) was higher than non-TBI (1.3%; *p* < 0.001). Consistent with previous literature, TBI was associated with incident hypertension (odds ratio [OR] = 1.46, 95% confidence interval [CI] = 1.19–1.78, *p* < 0.001). Notably, TBI was associated with an increased risk of ischemic stroke (OR = 3.16, 95% CI = 1.94–5.16, *p* < 0.001), as was hypertension (OR = 1.54, 95% CI = 1.04–2.28, *p* = 0.03).

**Table 1.** Baseline Characteristics

	***Control* (** *n* *** = 1848)***	***TBI* (** *n* *** = 3664)***
Sex		
Female	878 (47.5%)	1842 (50.3%)
Male	970 (52.5%)	1822 (49.7%)
Age (years)		
Median age (IQR)	44.1 (29.9–55)	45.2 (30–55)
18–40	760 (41.1%)	1367 (37.3%)
41–60	811 (43.9%)	1743 (68.2%)
>60	277 (15%)	554 (66.7%)
Race/ethnicity		
White	1481 (80.1%)	2858 (78%)
Black	146 (7.9%)	233 (6.4%)
Hispanic	113 (6.1%)	206 (5.6%)
Other^a^	108 (5.8%)	367 (10%)
First encounter setting		
Inpatient	186 (10.1%)	673 (18.4%)
Outpatient	1414 (76.5%)	2685 (73.3%)
Outpatient-emergency	7 (0.4%)	3 (0.1%)
Missing	241 (13%)	303 (8.3%)
Injury Severity Score, mean (SD)	—	6.4 (±4.4)
Follow-up after index encounter, median (IQR), years	7.9 (5.6–10)	8.7 (6–10)
Ischemic stroke		
Ischemic stroke	19 (1%)	136 (3.7%)
Mean age at ischemic stroke, year (SD)	61.2 (±14.5)	61.7 (±15.3)
Mean time from index date to ischemic stroke, year (SD)	5 (±1.6)	4.5 (±2.6)

^a^ Other includes Asian, Asian Pacific Islander, Hawaiian, Native American, and Middle Eastern.

IQR, interquartile range; SD, standard deviation; TBI, traumatic brain injury.

**FIG. 1.**
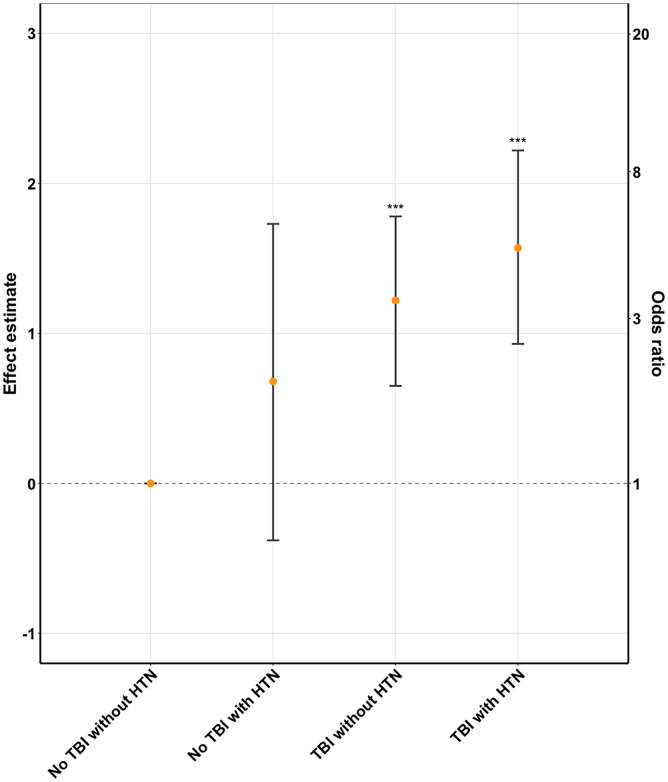
Effect estimates, odds ratios, and 95% confidence intervals for ischemic stroke by TBI and hypertension status. Patients without TBI or incident hypertension served as reference. Models adjusted for age, sex, race, and medical encounters. HTN, hypertension; TBI, traumatic brain injury

Examining the four exposure categories (no TBI nor hypertension as reference, hypertension only, TBI only, or both), those with TBI and hypertension were most likely to experience ischemic stroke (Fig. 1). However, the log odds estimate for ischemic stroke among patients with both incident hypertension and TBI (log odds_TBI+HTN_ = 1.57, 95% CI = 0.93–2.22, *p* < 0.001) was less than the sum of the log odds estimates for TBI only (log odds_TBI_ = 1.22, 95% CI = 0.65–1.78, *p* < 0.001) and incident hypertension only (log odds_HTN_ = 0.68, 95% CI = −0.38 to 1.73, *p* = 0.21).

To further investigate whether incident hypertension could serve as a potential mediator between TBI and ischemic stroke, we compared the TBI effect without hypertension (total effect) and with hypertension (CDE). The total effect of TBI (OR_TBI_ = 3.16, 95% CI = 1.94–5.16, *p* < 0.001) was higher in models that did not include incident hypertension when compared to the CDE of TBI (CDE OR_TBI_ = 2.45, 95% CI = 0.93–6.47, *p* = 0.06) in models that included hypertension status. When restricting the analysis to 553 patients who developed hypertension after the index date, the prevalence of ischemic stroke among those untreated with antihypertensives was 10.3% compared with 5.8% among those treated. The corresponding adjusted OR for the TBI effect was 2.72 (95% CI = 1.03–7.24, *p* = 0.04) among untreated and 1.88 (95% CI = 0.61–5.80, *p* = 0.27) among treated patients.

## Discussion

We prospectively compared the incidence of ischemic stroke in TBI and non-TBI patients who did and did not develop hypertension. Overall, our results are consistent with the hypothesis that incident hypertension may partially mediate the TBI effect on ischemic stroke based on the following observations: 1) The combined effect of concurrent TBI and hypertension was less than the sum of the individual effects; 2) the total effect of TBI was higher than the CDE that accounted for hypertension; and 3) use of antihypertensive medications reduced the effect of TBI on ischemic stroke among hypertensive patients. All the above suggest a potentially overlapping biological pathway that links TBI and incident hypertension with ischemic stroke.

Emerging data suggest that TBI is associated with increased risk of ischemic stroke,^1,6,7^ as is hypertension.^8^ Previously, the factors that mediate the effect of TBI on ischemic stroke had not been evaluated. Our data suggest that TBI's association with ischemic stroke may be partially mediated by TBI-precipitated hypertension. Proposed mechanisms linking TBI and ischemic stroke include autonomic dysregulation,^9^ neuroinflammation,^10^ accelerated atherosclerosis,^11^ and lifestyle changes post-TBI.^12^ If TBI and incident hypertension increase the risk of ischemic stroke along a shared biological pathway, that shared pathway could be attenuated by antihypertensive medication, as suggested by our data.

Although our data cannot identify causal relationships, results may have clinical implications. Given the lack of pre-existing cardiovascular comorbidities, results showing that incident hypertension may mediate TBI effects suggest that screening for and treating hypertension would not only reduce the risk of stroke attributable to hypertension directly, but may also reduce the risk of stroke attributable to TBI. Future work is needed to clarify the impact of hypertension management on ischemic stroke prevention in TBI patients. While we await these data, it may be prudent to specifically surveil TBI patients for hypertension beyond the acute phase of TBI and consider antihypertensives to reduce ischemic stroke arising from multiple contributing risks.

Study limitations include the following: Comorbidities were ascertained based on ICD codes, lack of data on ischemic stroke severity, hypertension severity, non-pharmacological management, medication types, and treatment response. The generalizability of these results may be limited because of an inadequate follow-up period. The low prevalence of hypertension reduced the statistical power to identify associations, and residual confounding may also be present in these data.

## Conclusion

In conclusion, our data offer evidence of an underpinning mechanism linking TBI with ischemic stroke, suggesting potentially overlapping biological mechanisms that may be responsive to pharmacological intervention. These results support the concept that TBI is a complex chronic multi-system disease that increases the risk of ischemic stroke.

## Supplementary Material

Supplemental data

## Transparency, Rigor, and Reproducibility Summary

This study followed the Strengthening the Reporting of Observational Studies in Epidemiology STROBE guidelines.^13^ The Mass General Brigham Institutional Review Board approved this study. The authors pre-specified the analysis plan. A hospital-based patient registry was queried to retrieve data spanning 2000–2018. A total of 8884 TBI patients were identified and frequency matched on age, sex, and race with 4442 non-TBI patients. After filtering for age ≥85 years and ≥4 years of follow-up, 3664 patients with TBI and 1848 non-TBI patients were included in the final analyses. Analyses were performed using R 4.2.2, with alpha defined as *p* < 0.05. The R codes used in this study are available upon request.

## Abbreviations Used

CDE: conditional direct effect
CI: confidence interval
ICD-9: International Classification of Diseases, Ninth Revision
ICD-10: International Classification of Diseases, Tenth Revision
OR: odds ratio
RPDR: Research Patient Data Registry
TBI: traumatic brain injury
