# Supplementary material for: Risk of Long-Term Ischemic Stroke in Patients With Traumatic Brain Injury and Incident Hypertension
Source: Neurotrauma Rep. 2024 Apr 22;5(1):462–6. doi: 10.1089/neur.2024.0015 (PMC11044850; doi:10.1089/neur.2024.0015)
Supplement: Supplemental data [file Suppl_TableS1.docx]

**Supplemental Table 1. ICD 9 and 10 codes used for comorbidity diagnoses for inclusion and exclusion criteria.**

| **Comorbidities** | **ICD-9** | **ICD-10** |
| --- | --- | --- |
| ***Traumatic brain injury*** | 800.0–801.9, 803.0–804.9, 850.0–854.1, 959.01, 807.0–807.9, 812–819.9, 822–822.9, or 823–827.9 | S06.0X0A, S06.0X1A, S06.0X9A, S02.0, S02.10, S02.91, S06.1X, S06.2X, S06.30-S06.38, S06.4-S06.9 |
| ***Excluded diagnoses:*** | | |
| ***Neurological disease*** | | |
| **Stroke** | 433, 434, 435, 436, 437 | I63, I65, I66, I67 |
|  | | |
| ***Cardiometabolic Disease*** | | |
| **Hypertension** | 401, 402, 403, 404, 405 | I10, I11, I12, I13, I15, I16 |
| **Hyperlipidemia** | 272 | E78 |
| **Obesity** | 278 | E66, Z68.25-Z68.44 |
| **Coronary artery disease** | 410, 411, 412, 413, 414 | I21, I22, I23, I24, I25 |
| **Diabetes mellitus** | 250, 790.29, 790.21 | E08, E09, E11, E13 |
|  |  |  |
| ***Psychiatric disease*** | | |
| **Depression** | 296.2, 296.3, 296.8, 300.4, 311 | F32, F33 |
| **Anxiety disorder** | 300, 300.2, 308, 309 | F41, F40.8, F40.9, F43.2 |
| **Substance use disorder** | 304.1-304.6, 304.8-304.9, 305.2, 305.3, 305.4, 305.6-305.9 | F12, F13, F14, F15, F16, F18, F19 |
| **Opioid use disorder** | 304.0, 304.7, 305.5 | F11 |
| **Alcohol use disorder** | 303, 305.0 | F10 |
